# A comprehensive machine-readable view of the mammalian cholesterol biosynthesis pathway

**DOI:** 10.1016/j.bcp.2013.03.021

**Published:** 2013-07-01

**Authors:** Alexander Mazein, Steven Watterson, Wei-Yuan Hsieh, William J. Griffiths, Peter Ghazal

**Affiliations:** a Division of Pathway Medicine, University of Edinburgh, Chancellor's Building, Little France Crescent, Edinburgh EH16 4SB, Scotland, UK; b Centre for Synthetic and Systems Biology, University of Edinburgh, CH Waddington Building, The King's Buildings, Mayfield Road, Edinburgh EH9 3JD, Scotland, UK; c Institute of Mass Spectrometry, College of Medicine, Grove Building, Swansea University, Singleton Park, Swansea SA2 8PP, Wales, UK

**Keywords:** Cholesterol, Sterol, SBGN, Metabolic network, Pathway map

## Abstract

Cholesterol biosynthesis serves as a central metabolic hub for numerous biological processes in health and disease. A detailed, integrative single-view description of how the cholesterol pathway is structured and how it interacts with other pathway systems is lacking in the existing literature. Here we provide a systematic review of the existing literature and present a detailed pathway diagram that describes the cholesterol biosynthesis pathway (the mevalonate, the Kandutch-Russell and the Bloch pathway) and shunt pathway that leads to 24(S),25-epoxycholesterol synthesis. The diagram has been produced using the Systems Biology Graphical Notation (SBGN) and is available in the SBGN-ML format, a human readable and machine semantically parsable open community file format.

## Graphical abstract

We present the pathways leading to cholesterol, epoxy-cholesterol and oxysterol synthesis.

**Figure:**
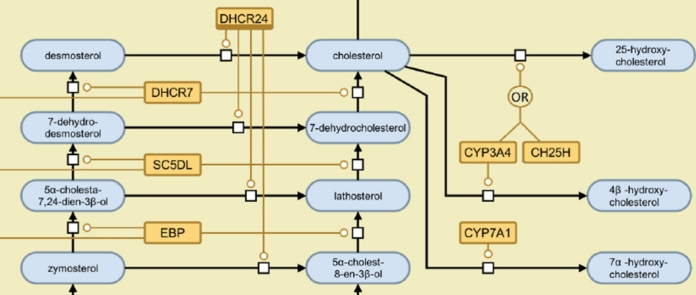

## 1 Introduction

Cholesterol is an intensively studied, multi-functional lipid that is key to many aspects of immunological, neuronal, viral and hepatocyte biology. It is an essential component of cellular membranes and is a precursor to steroids, bile acids and oxysterols whilst its own precursors contribute to prenylation and dolichylation and the formation of vitamin D_3_. One of the oxysterols known to be involved in linking sterol metabolism to innate immunity [1], [2] is 25-hydroxycholesterol. However its place in the sterol metabolism has not yet been well established.

Despite the importance of the cholesterol synthesis pathway to cellular function and its value in pharmaceutical therapies, an integrative picture of how the pathway is structured has not been well described in the literature, impeding the development of a more rigorous understanding of the role of the cholesterol metabolism in cellular processes. Publications typically focus on segments of the cholesterol biosynthesis pathway showing variable level of details. Kovacs and co-authors focus on the mevalonate section of the pathway and on the subcellular location of the enzymes involved [3]. Wang and co-authors concentrate on the steps leading to 24(S),25-epoxycholesterol synthesis and their similarity to steps in the cholesterol biosynthesis pathway [4]. Previous work studying the role of the cholesterol biosynthesis pathway has shown a modest level of detail on the sterol arms of the pathway [5], [6], [7] in innate immunity. The LIPID MAPS consortium offers the most detailed descriptions of the Bloch and Kandutsch-Russell branches of cholesterol biosynthesis, but these lack cell compartment information and lack integration with the 24(S),25-epoxycholesterol shunt arm and other branching pathways [8].

Here we present a comprehensive literature review of the cholesterol synthesis pathway and we implement this as a detailed pathway that captures enzymatic activity and compartmental localization and summarizes all intermediate metabolic forms. Our review also clearly indicates what information is missing and where additional research is required.

## 2 Materials and methods

The model of the cholesterol biosynthesis pathway presented in this work has been assembled using a variety of publicly available resources including the research findings of the LipidMaps consortium [8] and results obtained from thorough searches of the published literature that have been manually curated and validated by domain experts.

In cases where there were conflicting reports, preference was given to the more recent papers and to the works in which more reliable and advanced methods were used. The suggested order of events is supported by a number of independently obtained research results. The principles of the Evidence Ontology (ECO) [9], the Gene Ontology Evidence Codes [10] and the Evidence Code Decision Tree [11] were considered during the pathway reconstruction.

A brief summary is provided for each enzyme and the corresponding metabolic reactions involved in the pathway. For each enzyme we endeavored to capture the following information where available: corresponding gene name approved by HUGO Gene Nomenclature Committee [12], alternative names, enzymological activities according to the Enzyme Nomenclature Committee of the IUBMB [13], enzyme function description, subunit structure, subcellular location and related disorders.

We have included a list of UniProt IDs for the proteins captured in the model (Table 1) and a list of metabolite names (common and systematic) as used in the LipidMaps database [8] (Table 2). Common names are used on the map where available.

**Table 1:** Uniprot IDs for the enzymes in the pathways shown in Fig. 1, Fig. 2.

Uniprot ID	Link	EC	Order	Gene name	Protein name	Related disease	Reference
P24752	http://www.uniprot.org/uniprot/P24752	2.3.1.9	1	ACAT1	Acetyl-CoA acetyltransferase, mitochondrial; THIL	Alpha-methylacetoacetic aciduria	OMIM #203750
Q9BWD1	http://www.uniprot.org/uniprot/Q9BWD1	2.3.1.9	1	ACAT2	Acetyl-CoA acetyltransferase, cytosolic	ACAT2 deficiency	OMIM #614055
Q01581	http://www.uniprot.org/uniprot/Q01581	2.3.3.10	2	HMGCS1	Hydroxymethylglutaryl-CoA synthase, cytoplasmic	Not found	OMIM #142940
P54868	http://www.uniprot.org/uniprot/P54868	2.3.3.10	2	HMGCS2	Hydroxymethylglutaryl-CoA synthase, mitochondrial	HMG-CoA synthase deficiency	OMIM #605911; PMID 16601895
P35914	http://www.uniprot.org/uniprot/P35914	4.1.3.4	3	HMGCL	Hydroxymethylglutaryl-CoA lyase, mitochondrial	HMG-CoA lyase deficiency	OMIM #246450; PMID 17692550
P04035	http://www.uniprot.org/uniprot/P04035	1.1.1.34	4	HMGCR	3-Hydroxy-3-methylglutaryl-coenzyme A reductase	Not found	OMIM #142910
Q03426	http://www.uniprot.org/uniprot/Q03426	2.7.1.36	5	MVK	Mevalonate kinase	Mevalonic aciduria	OMIM #610377; PMID 16722536
Q15126	http://www.uniprot.org/uniprot/Q15126	2.7.4.2	6	PMVK	Phosphomevalonate kinase	Not found	OMIM #607622
P53602	http://www.uniprot.org/uniprot/P53602	4.1.1.33	7	MVD	Mevalonate diphosphate decarboxylase	Not found	OMIM #603236
Q13907	http://www.uniprot.org/uniprot/Q13907	5.3.3.2	8	IDI1	Isopentenyl-diphosphate delta-isomerase 1	Not found	OMIM #604055
Q9BXS1	http://www.uniprot.org/uniprot/Q9BXS1	5.3.3.2	8	IDI2	Isopentenyl-diphosphate delta-isomerase 2	Not found	Not found
P14324	http://www.uniprot.org/uniprot/P14324	2.5.1.10; 2.5.1.1	9	FDPS	Farnesyl diphosphate synthase; dimethylallyltranstransferase	Not found	Not found
O95749	http://www.uniprot.org/uniprot/O95749	2.5.1.29; 2.5.1.10; 2.5.1.1	10	GGPS1	Geranylgeranyl pyrophosphate synthase; farnesyl diphosphate synthase; dimethylallyltranstransferase	Not found	Not found
P37268	http://www.uniprot.org/uniprot/P37268	2.5.1.21	11	FDFT1	Farnesyl-diphosphate farnesyltransferase 1; squalene synthase	Not found	OMIM #184420
Q14534	http://www.uniprot.org/uniprot/Q14534	1.14.13.132	12	SQLE	Squalene monooxygenase; squalene epoxidase	Not found	OMIM #602019
P48449	http://www.uniprot.org/uniprot/P48449	5.4.99.7	13	LSS	Lanosterol synthase	Not found	OMIM #600909
Q15392	http://www.uniprot.org/uniprot/Q15392	1.3.1.72	14	DHCR24	Delta(24)-sterol reductase; 24-dehydrocholesterol reductase	Desmosterolosis	OMIM #602398; PMID 21559050
Q16850	http://www.uniprot.org/uniprot/Q16850	1.14.13.70	15	CYP51A1	Lanosterol 14-alpha demethylase; cytochrome P450, family 51, subfamily A, polypeptide 1	Not found	OMIM #601637
O76062	http://www.uniprot.org/uniprot/O76062	1.3.1.70	16	TM7SF2	Delta(14)-sterol reductase; transmembrane 7 superfamily member 2	Not found	OMIM #603414
Q15800	http://www.uniprot.org/uniprot/Q15800	1.14.13.72	17	MSMO1; SC4MOL	Methylsterol monooxygenase 1	Psoriasiform dermatitis; microcephaly; developmental delay	PMID 21285510
Q15738	http://www.uniprot.org/uniprot/Q15738	1.1.1.170	18	NSDHL	Sterol-4-alpha-carboxylate 3-dehydrogenase, decarboxylating; NAD(P) dependent steroid dehydrogenase-like	CK syndrome; CHILD syndrome	OMIM #300831; PMID 21129721; OMIM #308050
P56937	http://www.uniprot.org/uniprot/P56937	1.1.1.270	19	HSD17B7	3-Keto-steroid reductase; 17-beta-hydroxysteroid dehydrogenase 7	Not found	OMIM #606756
Q15125	http://www.uniprot.org/uniprot/Q15125	5.3.3.5	20	EBP	3-Beta-hydroxysteroid-delta(8),Delta(7)-isomerase; emopamil-binding protein	Conradi-Hünermann-Happle syndrome	OMIM #302960; PMID 21163155; PMID 22229330
O75845	http://www.uniprot.org/uniprot/O75845	1.14.21.6	21	SC5DL	Lathosterol oxidase; sterol-C5-desaturase (ERG3 delta-5-desaturase homolog, S. cerevisiae)-like	Lathosterolosis	OMIM #607330; PMID 12189593
Q9UBM7	http://www.uniprot.org/uniprot/Q9UBM7	1.3.1.21	22	DHCR7	7-Dehydrocholesterol reductase	Smith-Lemli-Opitz syndrome (SLOS)	OMIM #270400; PMID 23059950; PMID 23042628
P08684	http://www.uniprot.org/uniprot/P08684	1.14.13.97	23	CYP3A4	Cytochrome P450 3A4	Not found	OMIM #124010
O95992	http://www.uniprot.org/uniprot/O95992	1.14.99.38	24	CH25H	Cholesterol 25-hydroxylase	Alzheimer's disease	OMIM #604551; PMID 20580938; PMID 16909003
P22680	http://www.uniprot.org/uniprot/P22680	1.14.13.17	25	CYP7A1	Cholesterol 7-alpha-monooxygenase	Not found	OMIM #118455
Q02318	http://www.uniprot.org/uniprot/Q02318	1.14.13.15	26	CYP27A1	Sterol 26-hydroxylase, mitochondrial	Cerebrotendinous xanthomatosis	OMIM #606530; PMID 2019602; PMID 8514861
Q9Y6A2	http://www.uniprot.org/uniprot/Q9Y6A2	1.14.13.98	27	CYP46A1	Cholesterol 24-hydroxylase	Alzheimer's disease	OMIM #604087; PMID 12533085; PMID 12533083

**Table 2:** Systematics names, common names (where available) and LipidMaps IDs for the metabolites shown in Fig. 1, Fig. 2.

Pathway	LM_ID	Common Name	Systematic Name
Lanosterol biosynthesis	LMFA07050029	Acetyl-CoA	Acetyl-CoA
Lanosterol biosynthesis	LMFA07050030	Acetoacetyl-CoA	Acetoacetyl-CoA
Lanosterol biosynthesis	LMFA07050028	HMG-CoA	3S-Hydroxy-3-methyl-glutaryl CoA
Lanosterol biosynthesis	LMFA01050352	Mevalonic acid	3R-Methyl-3,5-dihydroxy-pentanoic acid
Lanosterol biosynthesis	LMFA01050415	Mevalonate-P	3R-Methyl-3-hydroxypentanoic acid 5-phosphate
Lanosterol biosynthesis	LMFA01050416	Mevalonate-PP	3R-Methyl-3-hydroxypentanoic acid 5-diphosphate
Lanosterol biosynthesis	LMPR01010008	Isopentenyl-diphosphate	3-Methylbut-3-enyl pyrophosphate
Lanosterol biosynthesis	LMPR01010001	Dimethylallyl-diphosphate	3-Methylbut-2-enyl pyrophosphate (dimethylallyl-diphosphate)
Lanosterol biosynthesis	LMPR0102010001	Geranyl diphosphate	Geranyl pyrophosphate
Lanosterol biosynthesis	LMPR0103010002	Farnesyl diphosphate	Farnesyl pyrophosphate
Lanosterol biosynthesis	LMPR0106010003	Presqualene diphosphate	Presqualene diphosphate
Lanosterol biosynthesis	LMPR0106010002	Squalene	Squalene
Lanosterol biosynthesis	LMPR0106010010	3S-Squalene-2,3-epoxide	2,3S-Epoxy-2,6,10,15,19,23-hexamethyltetracosa-6E,10E,14E,18E,22-pentaene
Lanosterol biosynthesis	LMST01010017	Lanosterol	Lanosta-8,24-dien-3β-ol
Cholesterol biosynthesis (Bloch)	LMST01010124	32-Hydroxylanosterol	4,4-Dimethyl-14α-hydroxymethyl-5α-cholesta-8,24-dien-3β-ol
Cholesterol biosynthesis (Bloch)	LMST01010222	32-oxolanosterol	4,4-Dimethyl-14α-formyl-5α-cholesta-8,24-dien-3β-ol
Cholesterol biosynthesis (Bloch)	LMST01010149	4,4-dimethylcholesta-8,11,24-trienol	4,4-Dimethyl-5α-cholesta-8,14,24-trien-3β-ol
Cholesterol biosynthesis (Bloch)	LMST01010176	14-demethyllanosterol	4,4-Dimethyl-5α-cholesta-8,24-dien-3β-ol
Cholesterol biosynthesis (Bloch)	LMST01010232	not available	4α-Hydroxymethyl-4β-methyl-5α-cholesta-8,24-dien-3β-ol
Cholesterol biosynthesis (Bloch)	LMST01010229	not available	4α-Formyl-4β-methyl-5α-cholesta-8,24-dien-3β-ol
Cholesterol biosynthesis (Bloch)	LMST01010150	4α-carboxy-4β-methyl-zymosterol	4α-Carboxy-4β-methyl-cholesta-8,24-dien-3β-ol
Cholesterol biosynthesis (Bloch)	LMST01010237	3-Keto-4α-methyl-zymosterol	4α-Methyl-5α-cholesta-8,24-dien-3-one
Cholesterol biosynthesis (Bloch)	LMST01010151	4α-Methyl-zymosterol	4α-Methyl-5α-cholesta-8,24-dien-3β-ol
Cholesterol biosynthesis (Bloch)	LMST01010234	Not available	4α-Hydroxymethyl-5α-cholesta-8,24-dien-3β-ol
Cholesterol biosynthesis (Bloch)	LMST01010226	Not available	4α-Formyl-5α-cholesta-8,24-dien-3β-ol
Cholesterol biosynthesis (Bloch)	LMST01010152	4α-Carboxy-zymosterol	4α-Carboxy-5α-cholesta-8,24-dien-3β-ol
Cholesterol biosynthesis (Bloch)	LMST01010168	Zymosterone	5α-Cholesta-8,24-dien-3-one
Cholesterol biosynthesis (Bloch)	LMST01010066	Zymosterol	5α-Cholesta-8,24-dien-3β-ol
Cholesterol biosynthesis (Bloch)	LMST01010206	5α-Cholesta-7,24-dien-3β-ol	5α-Cholesta-7,24-dien-3β-ol
Cholesterol biosynthesis (Bloch)	LMST01010121	7-Dehydro-desmosterol	Cholest-5,7,24-trien-3β-ol
Cholesterol biosynthesis (Bloch)	LMST01010016	Desmosterol	Cholest-5,24-dien-3β-ol
Cholesterol biosynthesis (Kandustch-Russell)	LMST01010087	24,25-Dihydrolanosterol	5α-Lanost-8-en-3β-ol
Cholesterol biosynthesis (Kandustch-Russell)	LMST01010224	Not available	4,4-Dimethyl-14α-hydroxymethyl-5α-cholest-8-en-3β-ol
Cholesterol biosynthesis (Kandustch-Russell)	LMST01010223	Not available	4,4-Dimethyl-14α-formyl-5α-cholest-8-en-3β-ol
Cholesterol biosynthesis (Kandustch-Russell)	LMST01010277	Not available	4,4-Dimethyl-5α-cholesta-8,14-dien-3β-ol
Cholesterol biosynthesis (Kandustch-Russell)	LMST01010225	Not available	4,4-Dimethyl-5α-cholest-8-en-3β-ol
Cholesterol biosynthesis (Kandustch-Russell)	LMST01010233	Not available	4α-Hydroxymethyl-4β-methyl-5α-cholest-8-en-3β-ol
Cholesterol biosynthesis (Kandustch-Russell)	LMST01010230	Not available	4α-Formyl-4β-methyl-5α-cholest-8-en-3β-ol
Cholesterol biosynthesis (Kandustch-Russell)	LMST01010227	Not available	4α-Carboxy-4β-methyl-5α-cholest-8-en-3β-ol
Cholesterol biosynthesis (Kandustch-Russell)	LMST01010236	Not available	4α-Methyl-5α-cholest-8-en-3-one
Cholesterol biosynthesis (Kandustch-Russell)	LMST01010197	4α-Methylcholest-8-en-3β-ol	4α-Methyl-5α-cholest-8-en-3β-ol
Cholesterol biosynthesis (Kandustch-Russell)	LMST01010235	Not available	4α-Hydroxymethyl-5α-cholest-8-en-3β-ol
Cholesterol biosynthesis (Kandustch-Russell)	LMST01010231	Not available	4α-Formyl-5α-cholest-8-en-3β-ol
Cholesterol biosynthesis (Kandustch-Russell)	LMST01010228	Not available	4α-Carboxy-5α-cholest-8-en-3β-ol
Cholesterol biosynthesis (Kandustch-Russell)	LMST01010239	Not available	5α-Cholest-8-en-3-one
Cholesterol biosynthesis (Kandustch-Russell)	LMST01010096	Zymostenol	5α-Cholest-8-en-3β-ol
Cholesterol biosynthesis (Kandustch-Russell)	LMST01010089	Lathosterol	Cholest-7-en-3β-ol
Cholesterol biosynthesis (Kandustch-Russell)	LMST01010069	7-Dehydrocholesterol	Cholesta-5,7-dien-3β-ol
Cholesterol biosynthesis (Kandustch-Russell)	LMST01010001	Cholesterol	Cholest-5-en-3β-ol
Cholesterol biosynthesis (Kandustch-Russell/Bloch)	LMST01010018	25-Hydroxy-cholesterol	Cholest-5-en-3β,25-diol
Cholesterol biosynthesis (Kandustch-Russell/Bloch)	LMST01010014	4β-Hydroxy-cholesterol	Cholest-5-en-3β,4β-diol
Cholesterol biosynthesis (Kandustch-Russell/Bloch)	LMST01010013	7α-Hydroxy-cholesterol	Cholest-5-en-3β,7α-diol
Cholesterol biosynthesis (Kandustch-Russell/Bloch)	LMST01010057	27-Hydroxy-cholesterol	Cholest-5-en-3β,26-diol
Cholesterol biosynthesis (Kandustch-Russell/Bloch)	LMST01010164	24-Hydroxy-cholesterol	Cholest-5-en-3β,24-diol

**Fig. 1:**
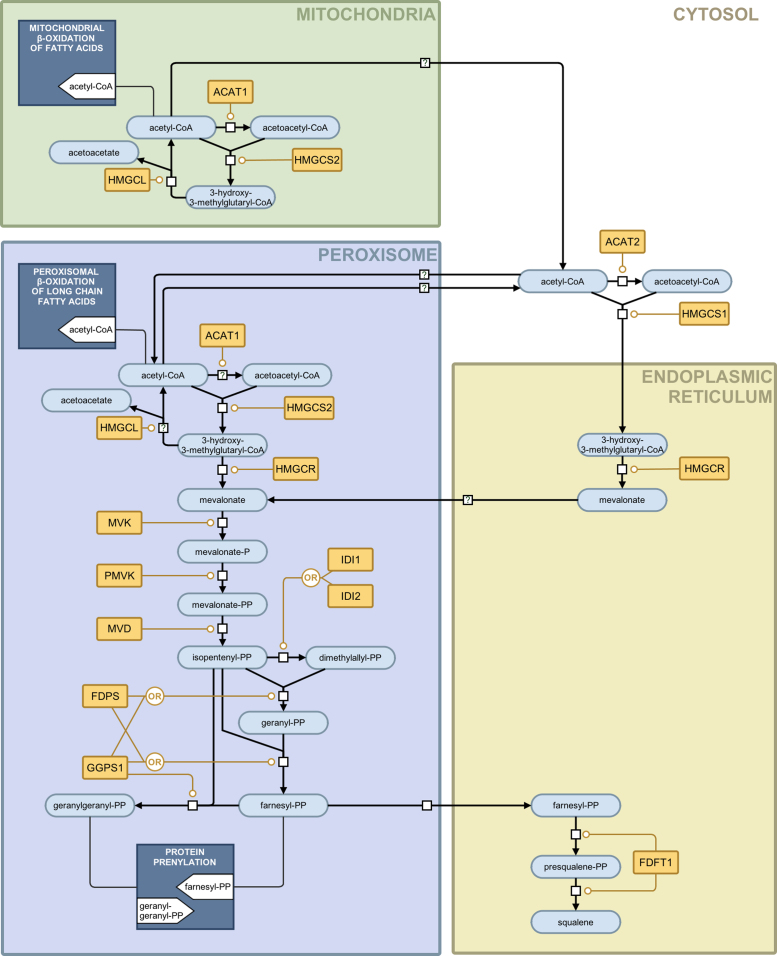
The mevalonate arm of the cholesterol biosynthesis pathway presented in SBGN notation. An interactive, parsable version of this figure, encoded using the SBGN-ML file format, is available in the supplementary material. The various glyph are explained in the legend in Fig. 3.

**Fig. 2:**
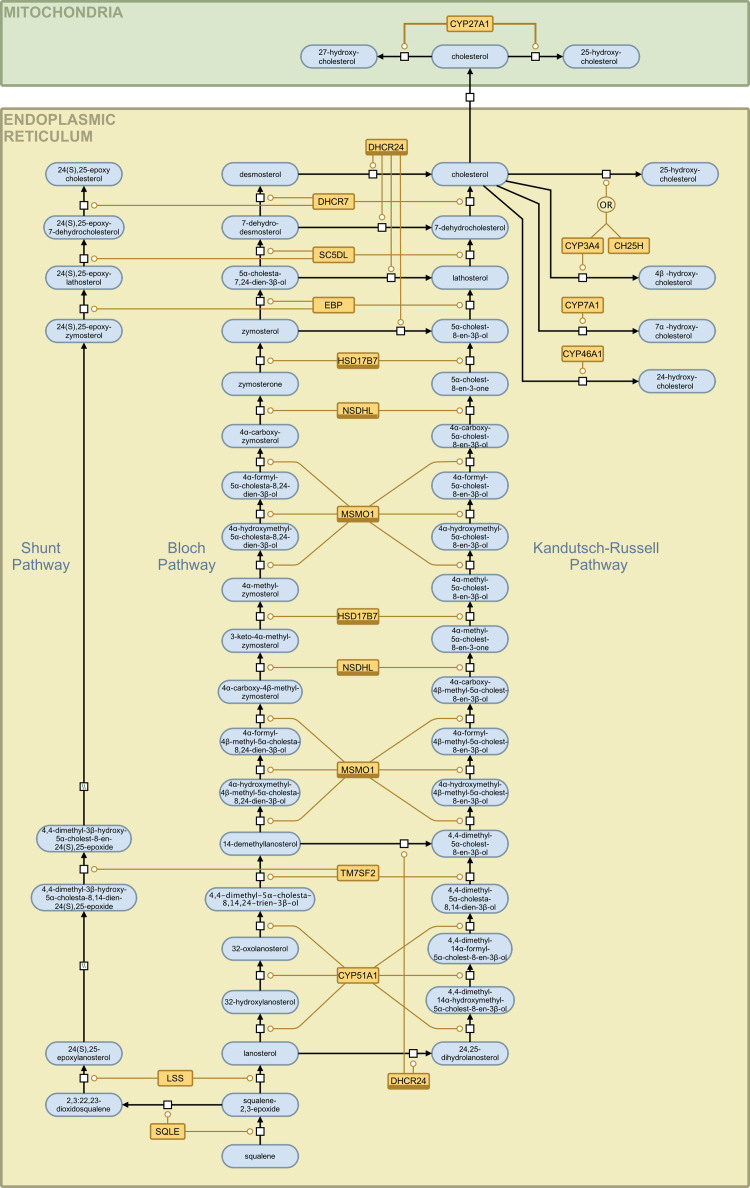
The sterol arms of the cholesterol biosynthesis pathway (shunt, Bloch and Kandutsch-Russell) presented in SBGN notation. An interactive, parsable version of this figure, encoded using the SBGN-ML file format, is available in the supplementary material. The various glyph are explained in the legend in Fig. 3.

**Fig. 3:**
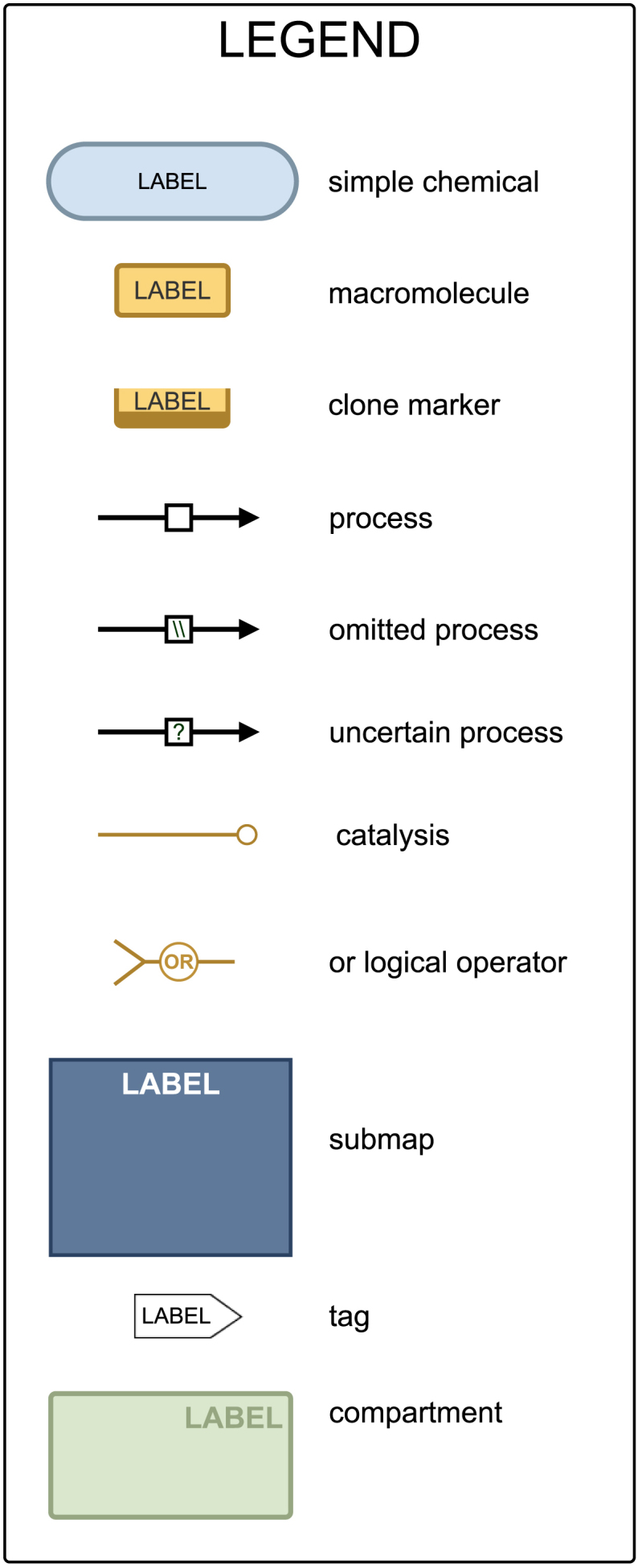
The legend explaining the SBGN glyphs used in Fig. 2, Fig. 3.

The pathway that we present here is described using the Systems Biology Graphical Notation (SBGN) [14], a community driven consensus graphical schema for capturing the molecular details of pathway systems. In particular, we use the SBGN Process Description language [15]. A machine-readable model is available as part of the supplementary material in SBGN-ML format [16] and we present it graphically in Fig. 1, Fig. 2, Fig. 3, in an enhanced form. The SBGN-ML format files can be read using a variety of software packages.

In particular, the supplementary files provide a description of the pathway that can be edited and modified in accordance with the SBGN standard in order to be of future use to the research community. The SBGN-ML file format encodes the biological meaning associated with each component of the model. This allows the model to be parsed by software (i) to ensure that modification is biologically valid and (ii) to facilitate automatic generation of mathematical descriptions of the pathway biology. It should be possible to open these files in any software designed to comply with the SBGN-ML standard, including but not limited to VANTED and Cytoscape [17], [18]. For the purpose of this review, we compiled and tested the files using the VANTED software tool [17]. Here we shall outline how the files can be opened and accessed using the VANTED and CYTOSCAPE [18] software tools.

### 2.1 Accessing supplementary SBGN-ML files using the VANTED software tool

***2.1.1*** Download the files with ‘.sbgn’ file extension from the supplementary material.
***2.1.2*** Download and install VANTED from http://vanted.ipk-gatersleben.de/.
***2.1.3*** Open VANTED and you will be greeted by a screen divided into two regions: an empty area on the left for diagrams and a column on the right containing settings under various tabs.
***2.1.4*** Using the automated installer to obtain the SBGN-ML add-on.
  ***2.1.4.1*** In the right hand column select the ‘Help’ tab and then the ‘Settings’ tab beneath and click on the ‘Install/Configure Add-ons’ button. This will open the Add-on Manager.
  ***2.1.4.2*** Click on the ‘Find Add-ons/Updates’ button on the bottom of the Add-on Manager window. This, in turn, opens the ‘Direct Add-on Download’ window.
  ***2.1.4.3*** At the top right of the Add-ons window, left and right arrows allow the user to move through a list of the available Add-ons. Find the Add-on entitled, ‘SBGN-ED’ and click the corresponding ‘Install Add-on’ button. VANTED will now automatically download the SBGN extension.
  ***2.1.4.4*** Click ‘OK’ and you will return to the ‘Add-on Manager’ where ‘SBGN-ED’ will now be listed as an Add-on. Ensure that the Active button is ticked beside the SBGN-ED entry to the list.
  ***2.1.4.5*** Click ‘OK’ on the ‘Add-on Manager’. The software is now installed.
  ***2.1.4.6*** From the menus at on the top of the VANTED window, select File>Open and choose your downloaded file with the ‘.sbgn’ file extension, in the usual way.
***2.1.5*** Manual installing the SBGN-ML add-on
  ***2.1.5.1** From*http://vanted.ipk-gatersleben.de/ Select Add-ons and then ‘SBGN-ED – Editing, Translating and Validating of SBGN Maps’.
  ***2.1.5.2*** Select ‘Download & Installation’ and then ‘SBGN-ED’ under downloads. A file called sbgn-ed.jar should start to download.
  ***2.1.5.3*** Return to Vanted and in the right hand column select the ‘Help’ tab and then the ‘Settings’ tab beneath. Click on the ‘Install/Configure Add-ons’ button. This will open the Add-on Manager.
  ***2.1.5.4*** Click the ‘Install Add-on’ button and select the sbgn-ed.jar file downloaded previously. Click the ‘Install’ button. This will return you to the ‘Add-on Manager’.
  ***2.1.5.5*** A message will appear the top of the Add-on Manager window stating that ‘Add-on “sbgn-ed.jar” will be updated when application is restarted’. Select OK and quit the program, before relaunching it.
  ***2.1.5.6*** From the menus at on the top of the VANTED window, select File>Open and choose your downloaded file with the ‘.sbgn’ file extension, in the usual way.

### 2.2 Accessing supplementary SBGN-ML files using the Cytoscape software tool

**2.2.1** Download and install Cytoscape from http://www.cytoscape.org/.
**2.2.2** Open Cytoscape and select the Plugins menu followed by ‘Manage Plugins’.
**2.2.3** In the search bar, type sbgn and hit return. Folders will appear in the window and under ‘Available for install’ will appear a Utility folder.
**2.2.4** Open the utility folder and select the latest version of CySBGN before hitting the install button. The CySBGN plugin will then be downloaded and installed. Once it is installed, close the ‘Manage Plugins’ window.
**2.2.5** From the File menu select import followed by ‘Network (Multiple File Types)’. In the window that opens, make sure that the ‘Local’ option is chosen and high the ‘Select’ button to bring up a file selector. Choose the downloaded file with the ‘sbgn’ file extension in the usual way.

## 3 Results and discussion

### 3.1 Pathway maps

Fig. 1 shows the mevalonate arm of the cholesterol biosynthesis pathway and includes enzymatic activity in the mitochondria, peroxisome, cytoplasm and endoplasmic reticulum. The arm starts with the consumption of acetyl-CoA, which occurs in parallel in three cell compartments (the mitochondria, cytoplasm and peroxisome) and terminates with the production of squalene in the endoplasmic reticulum. Fig. 2 shows the sterol arms of the cholesterol biosynthesis pathway and this includes the Bloch pathway, the Kandutsch-Russell pathway and the shunt pathway. This arm starts with Squalene and terminates with cholesterol production on the Bloch and Kandutsch-Russell pathways and with 24(S),25-epoxycholesterol on the shunt pathway. Fig. 3 provides a legend for the SBGN schema, explaining the various nodes and edges.

### 3.2 Mevalonate arm of the cholesterol biosynthesis pathway

**3.2.1. Acetyl-CoA acetyltransferase (ACAT1; ACAT2;*****Acetoacetyl-CoA thiolase*****; EC 2.3.1.9)** is an enzyme that catalyzes the reversible condensation of two molecules of acetyl-CoA and forms acetoacetyl-CoA. This reaction is an important step in ketone body formation. Both mitochondrial ACAT1 and cytosolic ACAT2 enzymes are homotetramers [19], [20]. Kovacs et al. suggest a possibility of distribution of ACAT1 between peroxisomes and mitochondria as experimental evidence supports the formation of acetoacetyl-CoA in peroxisomes [3]. The proposed step in peroxisomes is shown in Fig. 1 by a reaction glyph with a question mark. Mutations of the ACAT1 gene cause alpha-methylacetoacetic aciduria, an autosomal recessive disorder [21].

**3.2.2.*****Hydroxymethylglutaryl-CoA synthase*****(HMGCS1; HMGCS2; EC 2.3.3.10)** forms HMG-CoA from acetyl-CoA and acetoacetyl-CoA. The two proteins with this enzymological activity are HMGCS1 and HMGCS2 (Table 1). HMGCS1 is a cytoplasmic enzyme and HMGCS2 is localized to mitochondria and peroxisome [3]. Ortiz and co-authors provide evidence for the involvement of HMGCS2 in producing cholesterol-convertible HMG-CoA [22]. Peroxisomal localization of this enzyme was subsequently confirmed and the significance of the peroxisomal pathway in cholesterol production was demonstrated [39], [40]. The schema proposed by Kovacs and co-authors implies that the mitochondrial component of HMG-CoA is being converted into acetyl-CoA and acetoacetate by HMGCL (see 3.2.3) and is not likely to be involved in further steps contributing to cholesterol formation [3]. The possibility of HMG-CoA transport from the mitochondria to the endoplasmic reticulum or peroxisome requires further study.

**3.2.3.*****Hydroxymethylglutaryl-CoA lyase, mitochondrial*****(HMGCL; EC 4.1.3.4)** is a key enzyme in the ketone body formation pathway that provides fuel to extrahepatic tissues [23]. It transforms HMG-CoA into acetyl-CoA and acetoacetate. HMGCL is a mitochondrial enzyme and Kovacs et al. suggest peroxisomal localization in addition to mitochondrial [3]. Since the peroxisomal localization is not confirmed yet, we show this step with a question mark on the diagram (Fig. 1). The enzyme deficiency (HMGCLD) or hydroxymethylglutaric aciduria may be due to a variety of mutations and can be fatal [24].

**3.2.4. 3-Hydroxy-3-methylglutaryl-coenzyme A reductase (HMGCR; EC 1.1.1.34)** catalyzes the conversion of 3-hydroxy-3-methylglutaryl-CoA into mevalonic acid. The enzyme is highly regulated by relevant signaling pathways that include the SREBP pathway [25]. Kovacs et al. confirm endoplasmic reticulum localization of HMGCR and provide evidence that suggests peroxisomal localization [3]. In our model both locations for this enzyme are included. This enzyme is conventionally regarded as being rate limiting in the pathway and its interactions are targeted by the statin class of drug.

**3.2.5. Mevalonate kinase (MVK; ATP:mevalonate 5-phosphotransferase; EC 2.7.1.36)** catalyzes conversion of mevalonate into phosphomevalonate. According to Hogenboom and co-authors [26], [27], [28], [29] mevalonate kinase (MVK), phosphomevalonate kinase (PMK) and mevalonate pyrophosphate decarboxylase (MVD) are cytosolic enzymes. This contradicts studies of Kovacs and co-authors that confirm their previous findings of peroxisomal localization of the three enzymes [30] using stable isotopic techniques and human cells [3]. MVK is regulated by intermediates of the cholesterol metabolism pathway [31]. MVK can be competitively inhibited by farnesyl- and geranyl-pyrophosphates [32]. Mutation of the MVK gene causes mevalonate kinase deficiency [33], a disorder that leads to the lower activity of the enzyme and the accumulation of mevalonic acid, resulting in mevalonic aciduria [34] and hyperimmunoglobulinemia D syndrome [35].

**3.2.6. Phosphomevalonate kinase (PMVK; EC 2.7.4.2)** catalyzes formation of mevalonate 5-diphosphate from mevalonate 5-phosphate, an essential step in the mevalonate pathway. It is a reversible reaction and kinetic constants have been determined for human enzymes, both for forward and reverse reactions [36], [37]. Expression of this enzyme is regulated in response to dietary sterol levels and this regulation is coordinated with HMGCR [38]. Peroxisomal localization of the enzyme has been confirmed [3], [30], [38], [39], [40].

**3.2.7. Diphosphomevalonate decarboxylase (MVD; mevalonate (diphospho) decarboxylase; EC 4.1.1.33** is an enzyme that decarboxylates mevalonate 5-diphosphate forming isopentenyl diphosphate while hydrolyzing ATP. This enzyme is considered to be a useful target for lowering serum cholesterol levels [41] and is active as a homodimer [41]. Information on peroxisomal localization of diphosphomevalonate decarboxylase is provided in the section on mevalonate kinase (3.2.5).

**3.2.8. Isopentenyl-diphosphate delta-isomerases (IDI1; IDI2; EC 5.3.3.2)** perform isomerization of isopentenyl diphosphate into dimethylallyl diphosphate. These metabolites serve as fundamental building blocks of isoprenoids. This is an essential rate-limiting regulatory step for isoprenoid biosynthesis [42]. There are two types of isopentenyl-diphosphate delta-isomerase, IDI1 and IDI2. They differ in their structure and activity [43]. Both enzymes act via a proton addition/elimination mechanism [44]. IDI2 requires the presence of a reduced flavin mononucleotide cofactor [43] and both enzymes are localized to the peroxisome [45].

**3.2.9. Farnesyl diphosphate synthase (FDPS; EC 2.5.1.10; EC 2.5.1.1; Dimethylallyltranstransferase)** catalyzes two reactions that lead to farnesyl diphosphate formation. In the first (EC 2.5.1.1 activity) isopentenyl diphosphate and dimethylallyl diphosphate are transformed into geranyl diphosphate. Next, geranyl diphosphate and isopentenyl diphosphate are transformed into farnesyl diphosphate (EC 2.5.1.10 activity). The enzyme is a homodimer [46]. It is reported to be localized in peroxisomes [47]. FDPS supplies precursors for synthesis of steroids, dolichols and ubiquinones, protein fanesylation and geranylation. This enzyme has been suggested as an important target for drug development [48].

**3.2.10. Geranylgeranyl pyrophosphate synthase (GGPS1; EC 2.5.1.29; EC 2.5.1.10; farnesyl diphosphate synthase; EC 2.5.1.1; dimethylallyltranstransferase)** is able to catalyze the two reactions of farnesyl diphosphate formation. In addition, the enzyme catalyzes the addition of three molecules of isopentenyl diphosphate to dimethylallyl diphosphate and forms geranylgeranyl diphosphate, an important precursor of geranylated proteins [49] (EC 2.5.1.29 activity). The active enzyme is homohexamer [49] and is assumed to be localized to the endoplasmic reticulum.

**3.2.11. Farnesyl-diphosphate farnesyltransferase 1 (FDFT1; EC 2.5.1.21; Squalene synthase)** catalyzes a two-step reductive dimerization of two farnesyl diphosphate molecules and synthesizes squalene [50], [51], [52]. The FDFT1 expression level is regulated by cholesterol status: the human FDFT1 gene has a complex promoter with multiple binding sites for SREBP-1a and SREBP-2 [53].

### 3.3 Sterol arms of the sterol biosynthesis pathway

**3.3.1. Squalene epoxidase (SQLE; EC 1.14.13.132; squalene monooxygenase)** catalyzes the conversion of squalene into squalene-2,3-epoxide and the conversion of squalene-2,3-epoxide (2,3-oxidosqualene) into 2,3:22,23-diepoxysqualene (2,3:22,23-dioxidosqualene). The first reaction is the first oxygenation step in the cholesterol biosynthesis pathway and the second reaction is the first step in 24(S),25-epoxycholesterol formation from squalene-2,3-epoxide [4], [54]. The steps are localized to the endoplasmic reticulum membrane [55] and it has been suggested that this is one of the rate-liming steps in the pathway [56].

**3.3.2. Lanosterol synthase (LSS; OLC; OSC; 2,3-oxidosqualene:lanosterol cyclase; EC 5.4.99.7)** catalyzes cyclization of squalene-2,3-epoxide to lanosterol and 2,3:22,23-diepoxysqualene to 24(S),25-epoxylanosterol [4], [54]. The active monomeric enzyme is localized to the endoplasmic reticulum membrane [57]. Together with FDFT1 and SQLE, LSS has been considered as prospective target for antihypercholesterolemia drugs as an alternative to statin-based therapies [58], [59].

**3.3.3. Delta(24)-sterol reductase (DHCR24; 24-dehydrocholesterol reductase; EC 1.3.1.72)** is a special enzyme in cholesterol biosynthesis pathway due to its broad substrate specificity. It catalyzes the reduction of the delta-24 double bond of intermediate metabolites. In particular, DHCR24 converts lanosterol into 24,25-dihydrolanostrol, the initial metabolite of the Kandutsch-Russell pathway, and also provides the last step of Bloch pathway converting desmosterol into cholesterol. Intermediates of the Bloch pathway are converted by DHCR24 into intermediates of Kandutsch-Russell pathway [4]. Endoplasmic reticulum membrane localization has been confirmed for the enzyme [60]. Recent studies investigating the regulation of the DHCR24 promoter present evidence of binding sites for SREBP-2 [61], [62]. DHCR24 participates in the inflammatory response and induces heme oxigenase-1, a potentially cardioprotective enzyme [63]. Mutations in the DHCR24 gene causes desmosterolosis [64], a rare autosomal disorder that is characterized by elevated level of desmosterol in tissues [65].

**3.3.4. Lanosterol 14-alpha demethylase (CYP51A1; cytochrome P450, family 51, subfamily A, polypeptide 1; EC 1.14.13.70)** converts lanosterol into 4,4-dimethyl-5α-cholesta-8,14,24-trien-3β-ol and 24,25-dihydrolanosterol into 4,4-dimethyl-5α-cholesta-8,14-dien-3β-ol in three steps [8].

**3.3.5. Delta(14)-sterol reductase (TM7SF2; transmembrane 7 superfamily member 2; EC 1.3.1.70)** catalyzes reactions on the three branches of the cholesterol and 24(S),25-epoxycholesterol pathways [4], [8]. This enzyme is localized to the endoplasmic reticulum membrane [66].

**3.3.6. Methylsterol monooxygenase 1 (MSMO1; SC4MOL;*****C-4 methylsterol oxidase*****; EC 1.14.13.72)** catalyzes demethylation of C4-methylsterols [8]. This protein is localized to the endoplasmic reticulum membrane [67]. Mutations in the MSMO1 gene cause psoriasiform dermatitis, microcephaly and developmental delay [68].

**3.3.7. Sterol-4-alpha-carboxylate 3-dehydrogenase, decarboxylating (NSDHL; NAD(P) dependent steroid dehydrogenase-like; EC 1.1.1.170)** participates in several steps of post-squalene cholesterol and 24(S),25-epoxycholesterol synthesis [4], [8]. Defects in the NSDHL gene cause CK syndrome [69], recessive mental retardation syndrome [70] and CHILD syndrome [71], congenital hemidysplasia with ichythyosiform erythrodema and limb defects [72].

**3.3.8. 3-keto-steroid reductase (HSD17B7; 17-beta-hydroxysteroid dehydrogenase 7; EC 1.1.1.270)** was the last unknown enzyme of mammalian cholesterol biosynthesis [73]. It was previously reported in the regulation of the activity of sex steroids [4], [8], [73], [74]. It converts zymosterone into zymosterol in the Bloch pathway.

**3.3.9. 3-Beta-hydroxysteroid-delta(8),delta(7)-isomerase (EBP; emopamil-binding protein; EC 5.3.3.5)** catalyzes the conversion of delta(8)-sterols into delta(7)-sterols. Mutations in the EBP gene cause Conradi-Hunermann-Happle syndrome [75] characterized by punctiform calcification in the bones [76], [77]. It is an endoplasmic reticulum membrane localized protein [78].

**3.3.10. Lathosterol oxidase (SC5DL; Sterol-C5-desaturase (ERG3 delta-5-desaturase homolog, S. cerevisiae)-like; EC 1.14.21.6)** catalyzes the production of 7-dehydrocholesterol, 7-dehydrodesmosterol and 24(S),25-epoxy-7-dehydrocholesterol [5]. Endoplasmic reticulum membrane localization has been suggested [79]. Lanosterol side chain amines were reported to be selective inhibitors for lathosterol oxidase [80] and together with other inhibitors for the post-squalene enzymes of the cholesterol biosynthesis pathway were suggested as potential targets for therapies to reduce the risk of cardiovascular disease [81]. Defects in the enzyme are the cause of lathosterolosis [82], an autosomal recessive disorder that is characterized by multiple congenital anomalies and liver disease [83].

**3.3.11. 7-Dehydrocholesterol reductase (DHCR7; EC 1.3.1.21)** catalyses reduction of the C7–C8 double bond of 7-dehydrocholesterol and formation of cholesterol. It also produces desmosterol from 7-dehydrodesmosterol [8] and 24(S),25-epoxycholesterol from 24(S),25-epoxy-7-dehydrocholesterol [4]. Mutations in the gene that encodes DHCR7 causes Smith-Lemli-Opitz syndrome, a recessively inherited autosomal disease [84].

**3.3.12. Cytochrome P450, family 3, subfamily A, polypeptide 4 (CYP3A4; 1,8-cineole 2-exo-monooxygenase; taurochenodeoxycholate 6α-hydroxylase; EC 1.14.13.97)** catalyses the hydroxylation of cholesterol leading to 25-hydroxycholesterol [85] and 4β-hydroxycholesterol [86], [87]. It is localized to the endoplasmic reticulum [88].

**3.3.13. Cholesterol 25-hydroxylase (CH25H; Cholesterol 25-monooxygenase; EC 1.14.99.38)** uses di-iron cofactors to catalyze the hydroxylation of cholesterol [89], [90], [91] to produce 25-hydroxycholesterol. Has the capacity to catalyze the transition of 24-hydroxycholesterol to 24,25-dihydroxycholesterol. It is an endoplasmic reticulum localized enzyme [89] and has been shown to have a role in innate immunity [1], [2]. Mutations have been associated with Alzheimer's disease [92], [93].

**3.3.14. Cytochrome P450, family 7, subfamily A, polypeptide 1 (CYP7A1; cholesterol 7-alpha-hydroxylase; EC 1.14.13.17)** responsible for introducing a hydrophilic moiety at position 7 of cholesterol to form 7α-hydroxycholesterol [94]. It is localized to the endoplasmic reticulum [95].

**3.3.15. Cytochrome P450, family 27, subfamily A, polypeptide 1 (CYP27A1; Sterol 27-hydroxylase; EC 1.14.13.15)** was the first hydroxylase to be isolated [96]. It catalyses the transition of mitochondrial cholesterol to 27-hydroxycholesterol [97] and 25-hydroxycholesterol [98], [99]. It is localized to the mitochondria [97] and mutations are associated with the recessive autosome disorder Cerebrotendinous Xanthomatosis [100].

**3.3.16. Cytochrome P450 46A1 (CYP46A1, cholesterol 24-hydroxylase, EC 1.14.13.98)** catalyses transformation of cholesterol into 24(S)-hydroxycholesterol. Localized to endoplasmic reticulum membrane [101], the enzyme is mainly expressed in brain tissue and is considered to be an important biomarker for neurodegenerative disorders [102], [103], [104], [105].

### 3.4 Oxysterol 24(S),25-epoxycholesterol synthesis from squalene

24(S),25-Epoxycholesterol is produced in a shunt pathway that is parallel to the two branches of the cholesterol synthesis pathway [4], [106], [107], [108]. The same set of enzymes is involved in the formation of cholesterol from 24,25-dihydrolanosterol in the Kandutsch-Russell pathway, desmosterol from lanosterol in the Bloch pathway and 24(S),25-epoxycholesterol from 24(S),25-epoxylanosterol in a shunt pathway [4]. Due to its importance in regulatory processes 24(S),25-epoxycholesterol is in the focus of several recent publications [107], [109], [110], [111], [112]. However, further research is necessary to confirm each step of the shunt pathway and the corresponding intermediate metabolites. In our representation, the known intermediates of the shunt pathway are shown and likely missing information is noted with the appropriate SBGN glyph.

### 3.5 Transport of the intermediate metabolites between different cellular compartments

Little is known about transporting of the intermediate metabolites of the cholesterol biosynthesis pathway between different cellular compartments. It is not known whether metabolites in the mitochondria participate in cholesterol biosynthesis and, in a number of cases, it is unclear whether metabolites move between compartments through diffusion or transportation. This is something that should be addressed in the future. It has been suggested that limitations in subcellular fractionation methods are a significant factor in our poor understanding of the subcellular localization of these enzymes [3].

## 4 Concluding remarks

The diagrams presented here show a comprehensive view of the mevalonate, Kandutsch-Russell, Bloch and shunt pathways. The diagrams are described using the SBGN schema, an open and community developed graphical language for unambiguously capturing pathway structure. Missing/uncertain information is clearly marked on the pathway diagrams and shows the areas that need to be further explored. Models of these diagrams are available as supplementary material in the SBGN-ML and SBML/CellDesigner [113] file formats for future development and refinement.

We hope that by elucidating and integrating the detailed structure of this pathway, we will contribute to a finer level of understanding of cholesterol metabolism and its function and that this will serve as a useful resource for future studies of the cholesterol biosynthesis pathway.
